# Frankincense: A neuronutrient to approach Parkinson’s disease treatment

**DOI:** 10.1515/med-2024-0988

**Published:** 2024-06-17

**Authors:** Vittorio Calabrese, Naomi Osakabe, Foziya Khan, Uwe Wenzel, Sergio Modafferi, Lidia Nicolosi, Tilman Fritsch, Ursula M. Jacob, Ali S. Abdelhameed, Luay Rashan

**Affiliations:** Department of Biomedical and Biotechnological Sciences, University of Catania, Torre Biologica, 95125 Catania, Italy; Department of Bioscience and Engineering, Shibaura Institute Technology, Tokyo, Japan; Biodiversity Unit, Dhofar University, Salalah, Oman; Institute of Nutritional Science, Justus Liebig University of Giessen, Giessen, Germany; NAM Institute, 5020 Salzburg, Austria; System Biologie AG, Wollerau [CH], Switzerland; Department of Pharmaceutical Chemistry, College of Pharmacy, King Saud University, Riyadh 11451, Saudi Arabia

**Keywords:** antioxidants, hormesis, Parkinson’s disease, vitagenes

## Abstract

Parkinson’s disease (PD), characterized by tremor, slowness of movement, stiffness, and poor balance, is due to a significant loss of dopaminergic neurons in the substantia nigra pars compacta and dopaminergic nerve terminals in the striatum with deficit of dopamine. To date the mechanisms sustaining PD pathogenesis are under investigation; however, a solid body of experimental evidence involves neuroinflammation, mitochondrial dysfunction, oxidative stress, and apoptotic cell death as the crucial factors operating in the pathogenesis of PD. Nutrition is known to modulate neuroinflammatory processes implicated in the pathogenesis and progression of this neurodegenerative disorder. Consistent with this notion, the Burseraceae family, which includes the genera *Boswellia* and *Commiphora*, are attracting emerging interest in the treatment of a wide range of pathological conditions, including neuroinflammation and cognitive decline. Bioactive components present in these species have been shown to improve cognitive function and to protect neurons from degeneration in *in vitro*, animal, as well as clinical research. These effects are mediated through the anti-inflammatory, antiamyloidogenic, anti-apoptotic, and antioxidative properties of bioactive components. Although many studies have exploited possible therapeutic approaches, data from human studies are lacking and their neuroprotective potential makes them a promising option for preventing and treating major neurodegenerative disorders.

## 1 Neuronutrition and Parkinson’s disease (PD)

It is widely recognized that a progressive deterioration of physiological functions and metabolic processes underlies the pathogenic determinism of aging [1,2,3,4]. Thus, one of prominent idea of modern society remains the concept of healthy aging. As a disease associated with the elderly, in PD patients, a significant neuronal loss is the consequence of multifactorial events, primarily overproduction of reactive oxygen species (ROS) by mitochondria causing DNA oxidation and damage, apoptosis, and ferroptotic death of dopaminergic neurons [5,6,7,8]. In these settings, no efficient clinical treatment has been developed yet; however, increasing evidence indicates that nutritional antioxidants can delay the onset of disorders associated with aging, in spite of absence of active anti-aging drugs available. Once neurodegeneration begins, it can only be slowed but not stopped, emphasizing the clinical relevance of the disease.

After Alzheimer’s disease (AD), PD – which shows an increased frequency after the age of 50 years – represents the second most prevalent form of neurodegeneration, with selective loss of dopaminergic neurons in the pars compacta substantia nigra and significant drop of striatal dopamine content responsible for major clinical disturbances observed in this disease [5]. A large body of experimental evidence highlights a fundamental role of oxidative and nitrosative stress in the pathogenesis of PD [6], mainly due to the pro-oxidant action of catechols, including DOPA and dopamine, which undergo redox cycling processes generating cytotoxic products, primarily through formation of covalent bonds between their quinone redox active forms and important macromolecules, such as proteins and peptides where thiol groups are the primary target of oxidative attack [7]. Consistent with this notion, excitotoxic mechanisms are promoted by mitochondrially encoded defects occurring at levels of complex subunits, primarily complex I of the electron transport chain [8], which ensues in bioenergetic transduction defects, with impairment of oxidative phosphorylation, ATP, and glutathione depletion, and ultimately enhanced vulnerability to excitotoxic insults [9]. Studies from 1-methyl-4-phenyl-1,2,3,6-tetrahydropyridine neurotoxicity in mice as well as in primates have provided corroborating evidence that neuronal NO synthase inhibition induces neuroprotection, raising the conceivable possibility that the use of excitatory amino acid antagonists of neuronal NO synthase inhibitors might provide a rational for novel interventions in PD therapeutics [10].

**Figure 1:**
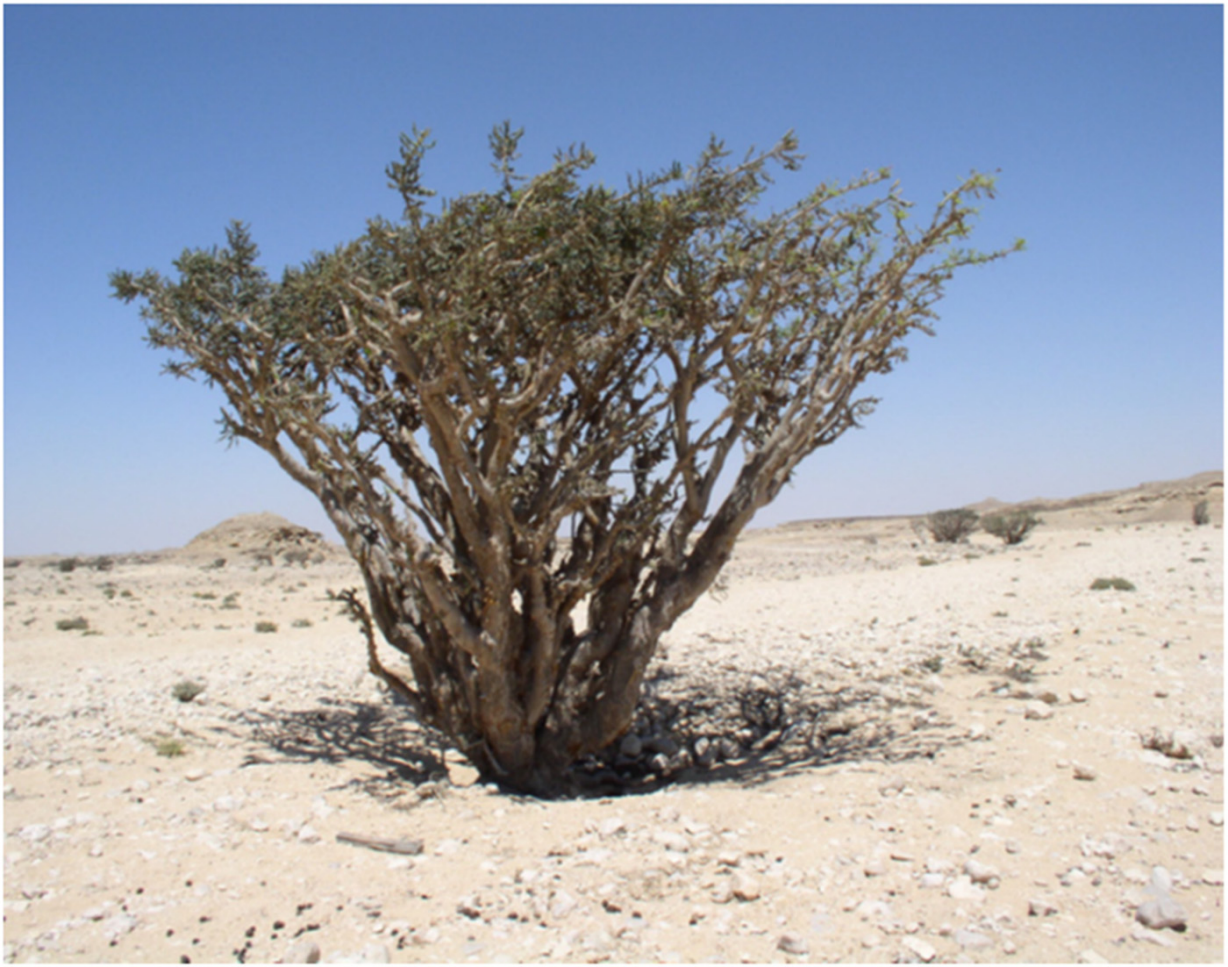
*Boswellia sacra* in Wadi Dawkah, a natural park of frankincense-producing trees in Oman.

**Figure 2:**
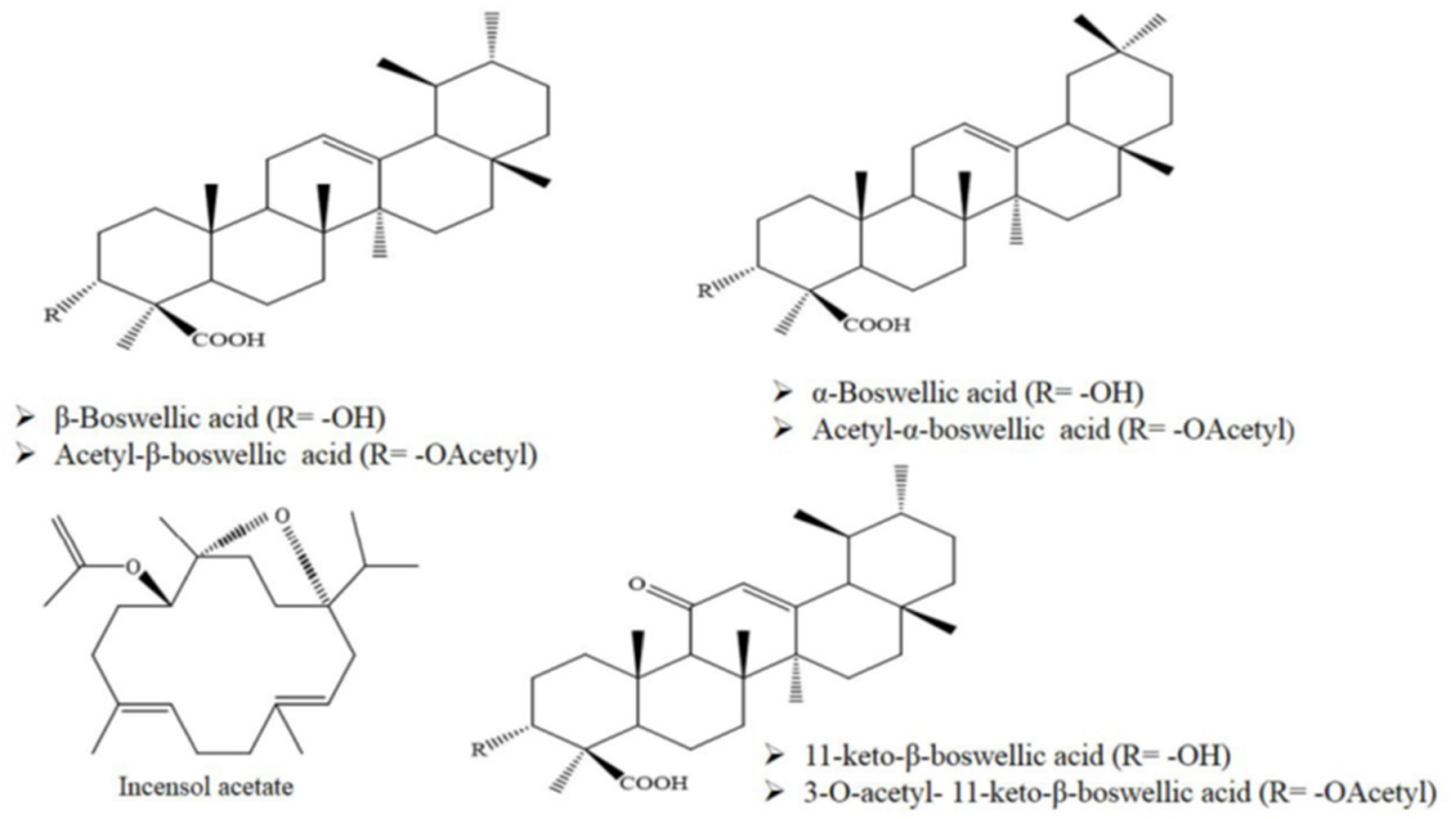
Chemical structure of main constituents of genus *Boswellia.*

Frankincense is derived from the *Boswellia sacra* Flück tree and represents a common source of traditional medicine. The word “frankincense” has got its origin from a very old French word, “franc encens,” which means “pure incense” or “pure and noble high-quality incense” [11]. The sweet aroma of smoldering frankincense gum has been used for centuries. The *Boswellia sacra* Flück tree, when cut at the base, exudes a toughened resin or gum-like substance [12]. Figure 1 illustrates the *B. sacra* tree, which belongs to the *Boswellia* genus. The *Boswellia* genus comprises 25 species that are widely distributed across various regions such as India, the Arabian Peninsula, North Africa, Somalia, Ethiopia, and Eritrea [13]. *Boswellia sacra* Flueck, one of the 25 species in the family *Boswellia* (*Burseraceae*), has been used for centuries as an incense ingredient due to its fragrant gums and resins. Southwest Oman, the Hadramawt, and the Mahra areas of Yemen are the only places on the Arabian Peninsula where we can find this native species [14,15]. Since before the dawn of recorded history, frankincense has been one of the most highly prized commodities [16]. Frankincense is known to be produced by several *Boswellia* species, mainly from *B. carterii* (Somalia), *B. sacra* (Oman), and *B. serrata* (India) [17]. Figure 2 illustrates the chemical structure of common boswellic acids found in frankincense. Literature data suggest that *Boswellia* resin possesses antioxidative properties, which is consistent with the presence of primary terpenic natural phenolic compounds, such as *cis*-Verbenol, Pinene, Hepthene trimethyl, and *trans*-Ocimeneare, as determined by gas chromatographic analysis, which were associated with a significant total phenol content (TPC), as well as to a 2, 2-diphenyl-1-picrylhydrazyl (DPPH) radical-scavenging activity [18,19]. Consistently, antioxidants such as limonene (22.4%) and esters such as duva-3,9,13-trien-1,5a-diol-1-acetate (21.4%) are the main compounds responsible for antioxidant activity [20]. Frankincense is composed of essential oil (5–9%), gum (20–23%), and resin (60%). Ingredients analysis to identify individual compounds showed a variety of bioactive substances including terpenoids, phenols, flavonoids, and phenylpropanoid [21]. The main active components isolated from oleo-gum resin of *B. serrata* are boswellic acid (BA), which possesses a wide range of biological and pharmacological effects, are endowed with neuroprotective activities [22,23,24]. BAs, pentacyclic triterpenic acids, show multiple biological effects, including anti-inflammation, antioxidative, and anti-excitotoxicity, all impacting therapeutically neurological disorders. In particular, neuroprotection induced by BAs is connected with an action on neurotoxic aggregates, which are decreased by BA treatment, an effect associated with decreased oxidative stress and amelioration of cognitive defects. Consistent with these notions, among the most important active principles within the multi-component mixture of *Boswellia serrata* resin, there is the pentacyclic triterpenoid Acetyl 11-keto-b-boswellic acid (AKBA). *Boswellia serrata resin extracts* (BAs) not only show an *in vivo* antioxidant activity in many pathological conditions, such as stroke, but also bowel disease, myocardial I/R injury, and pulmonary fibrosis. The neuroprotective potential of pentacyclic triterpenoid has recently gained increasing attention as, for instance, oleanolic acid shows protective effects on cerebral ischemic damage and H_2_O_2_-induced injury *in vitro*. Recently, it has been shown that a naturally occurring pentacyclic triterpenoid, ursolic acid, exerts neuroprotection in mice exposed to cerebral ischemia through the activation of Nrf2 pathway. Another study has provided experimental evidence that AKBA may have even a better antioxidant effect compared with ursolic acid. Based on these studies, AKBA, sharing similar chemical structure with ursolic acid, may promote the neuroprotection via the Nrf2 pathway [25]. Only recently, due to its action on proliferative processes, migration, metastasis, angiogenesis, and apoptosis promotion, BA has been proposed as interesting compound and effective agent against brain tumorigenesis [26]. Moreover, the antioxidative therapeutic potential from *Boswellia serrata* has been demonstrated in cerebro-vascular systems [26], as well as in mice in a rotenone-induced model of PD, where a significant attenuation of nigrostriatal dopaminergic neuronal loss has been demonstrated, an effect associated with reduced accumulation of α-synuclein and modulation of AMPK phosphorylation, as also shown in SHSY5 cells [27]. These results suggest that *Boswellia*-derived compounds protect dopaminergic neurons from rotenone neurotoxicity via activation of the AMPK signal and reduction in α-synuclein aggregation and associated neuroinflammation [22,28,29].

Recent investigations from our laboratory have provided compelling evidence that a single oral dose of 500 mg/kg *Boswellia sacra* administered 1 h after the trauma was capable of decreasing anxiety and altered locomotor activity, which are post-traumatic stress disorder markers, while ameliorating spatial learning and memory processes. When measuring biochemical markers of oxidative insult promoted by trauma-induced neuroinflammatory changes, we measured decreased levels of lipid peroxidation, as well as proinflammatory cytokines after *Boswellia* treatment, with parallel increase in antioxidant enzymes superoxide dismutase (SOD) and glutathione-Peroxidases. We also observed in the same experimental conditions that the increase in caspase-3, caspase-8, caspase-9, Bcl-2, and cytochrome C were diminished by *Boswellia* administration, with increases in Bcl2, indicating a significant regulatory action of apoptotic pathways [22,30].

### 1.1 *Caenorhabditis elegans* as a model for investigating the impact of redox modulators on PD

Since α-synuclein is considered to represent an amyloidogenic and pathogenic protein leading to the degeneration of dopaminergic neurons and the phenotype of PD, most of the *C. elegans* Parkinson´s models rely on the transgenic expression of α-synuclein under control of a dopaminergic or a pan-neuronal promoter [31,32]. Often, α-synuclein is fused to fluorescent proteins in order to enable the visualization of cellular degeneration as well [33]. However, in the absence of α-synuclein, specific labeling of dopaminergic neurons (two ADE and four CEP neurons in the head plus two PDE neurons in the posterior part of the body) by a fluorescent marker, which is expressed under the dopamine transporter promoter (Pdat-1), can serve the evaluation of dopaminergic degeneration. This has been exemplified by investigating the degeneration of dopaminergic neurons in response to the neurotoxin 6-hydroxydopamine (6-OHDA) in strains in which the dopaminergic forms of thioredoxin reductase or thioredoxin, i.e., trxr-1 and trx-5, were knocked out [34]. Increased degeneration of dopaminergic neurons in response to 6-OHDA was observed in the strain with trx-5 null alleles but even more pronounced in the strain with trxr-1 null alleles [34]. In a vicious cycle, redox imbalance is inevitably linked to mitochondrial dysfunction. In this context, it has been demonstrated that in *C. elegans* expressing α-synuclein in fusion with yellow fluorescent protein (YFP) under control of a muscle cell promoter, silencing of nuo-5 (linked to oxidative phosphorylation], F25B4.7 (shows ATP transmembrane transporter activity), and C05D11.9 (endowed with ribonuclease activity) led to increased α-synuclein aggregation and the attenuation of the mitochondrial quality control system, as exemplified by reduced transcript levels of a number of chaperones [35]. The most downregulated gene was hsp-60 [35], which suggests a decreased SOD-2 expression in association with increased mitochondrial oxidative stress and impaired respiration [36]. Using models that express α-synuclein fused to green fluorescent protein in dopaminergic neurons or fused to YFP transgenically overexpressed in body wall muscle cells, tyrosol, a polyphenol from extra virgin olive oil, was shown to reduce ROS and enhance the expression of chaperones and antioxidative enzymes [37]. Those effects were associated with reduced degeneration of dopaminergic neurons and ameliorated motility impairment in the strain expressing α-synuclein in muscle cells. Using the same *C. elegans* strain with the body wall muscle cell expression of α-synuclein, sesquiterpenoids from *Artemisia pallens* decreased the levels of aggregated α-synuclein [38]. One of them, bicyclogermacrene, increased the transcript levels of the SOD-1, SOD-2, and SOD-4 genes, encoding isoforms of the antioxidative enzyme, finally suggesting that the selected sesquiterpenoid acts through maintenance of cellular redox state and proteostasis (Table 1).

**Table 1:** Antioxidants present in Frankincense

Antioxidant	Results
Terpenes are natural phenolic compounds	Primary compounds in frankincense are *cis*-verbenol, pinene, hepthene trimethyl, and *trans*-Ocimeneare
Bilayer films of gelatin/frankincense with varying concentrations of Hyssopus officinalis and ascorbic acid	Significant changes in TPC and DPPH radical-scavenging activity
Other antioxidants	Limonene (22.4%) and esters such as duva-3,9,13-trien-1,5a-diol-1- acetate (1.4%)
Limonene, spathulenol, curzerene, beta selinene, isocericenine, myrcenol, and germacrene	Antioxidant and free radical scavenging properties and plays a significant role in fighting diseases that are related to the oxidative damage. Sesquiterpenoids have been found to possess antibacterial, antifungal, and anesthetic properties

## 2 Conclusion and future directions

Redox modulation of endogenous cellular resilience via cellular stress response signal pathway represents a novel convergent mechanism to approach therapeutically diseases associated with oxidative damage, such in neurodegenerative disorders [2]. Maintenance of repair processes at efficient functioning level is fundamental for brain cells survival and, consequently, overall quality of life. This is accomplished by a complex network of longevity assurance processes based on the expression and activity of redox regulated genes, termed vitagenes [39,40,41,42,43]. Consistent to this notion, upregulation of vitagenes likely results in a decreased occurrence of age-related disorders, with optimization of biochemical mechanisms underlying aging, as well as age-related processes. In this perspective, prolongation of a healthy life span may be a reachable clinical endpoint. For instance, in the United States with exponentially increasingly aged population, predictions indicate that the number of AD patients will reach 14 million in the mid-twenty-first century, if no effective interventions will be established [44,45,46,47]. This implies an immense economic and personal burden on USA nation welfare system, with similar conditions applying worldwide. Strong evidence exists to suggest that oxidative stress and impaired proteome, lipidome, and metabolome system networks sustain in a vicious cycle in the pathogenesis of major neurodegenerative disorders. With respect to this, brain-accessible redox active compounds, such as natural polyphenols, may represent a potentially active mean to implement therapeutic strategy limiting the onset of all degenerative diseases associated with oxidative stress, such as major brain neurological disorders. Moreover, vitagene system operates efficiently in the context of a longevity network which, by regulating hormetic nutrition processes in the whole organism, as well as at the level of single cells and tissues, by modulation of the energy status, regulate and optimize the functional state of mitochondria, mitochondrial ROS concentration, and ultimately coordinating biochemical information flow within signaling pathways, result in increasing life span. This highlights the central roles of HSF1 and Nrf2/keap1 neurobiology in stress resistance and proteostasis, whose enhanced activity during development and early adulthood is important for the stability of the proteome and the health of the cell across the entire lifespan. PD is a multifaceted disease with a convergence of multiple genetic, environmental, and cellular factors. Consistent with this evidence, while antioxidants and anti-inflammatory compounds may benefit, the disease’s complexity suggests that a single treatment approach may not address all aspects of PD pathogenesis, rather a multitargeted approach is warranted. In the present study, we evidence a lack of data from human studies, which is a significant limitation, as without robust clinical evidence, it is challenging to assess the true therapeutic potential of these compounds in humans. As we already started in our laboratory, clinical intervention trials are necessary to clearly elucidate the redox active therapeutic potential of these compounds impacting efficiently the disease pathogenesis, as well as the course of its progression. On the other hand, to date, we are not aware of negative impact on the neuroinflammatory component of PD pathogenesis either in animal-based models or in clinical studies. In addition, here we discuss the involvement of oxidative stress, mitochondrial dysfunction, and neuroinflammation, operating in a vicious cycle in the PD pathogenesis; however, it is important to note that the precise mechanisms by which these compounds exert their effects are not fully understood, and further research is needed to elucidate completely these mechanisms. More in general. while the potential benefits of *Boswellia*-derived compounds in PD are intriguing, more rigorous clinical studies are needed to establish their efficacy and safety in human populations. Additionally, a comprehensive understanding of their mechanisms of action and potential interactions with other PD therapies is crucial for their successful integration into PD multitargeted treatment strategies.
