# Corrigendum to “Frankincense: A neuronutrient to approach Parkinson’s disease treatment”

**DOI:** 10.1515/med-2024-2001

**Published:** 2024-12-19

**Authors:** Vittorio Calabrese, Naomi Osakabe, Foziya Khan, Uwe Wenzel, Sergio Modafferi, Lidia Nicolosi, Tilman Fritsch, Ursula M. Jacob, Ali S. Abdelhameed, Luay Rashan

**Affiliations:** Department of Biomedical and Biotechnological Sciences, University of Catania, Torre Biologica, 95125 Catania, Italy; Department of Bioscience and Engineering, Shibaura Institute Technology, Tokyo, Japan; Biodiversity Unit, Dhofar University, Salalah, Oman; Institute of Nutritional Science, Justus Liebig University of Giessen, Giessen, Germany; NAM Institute, 5020 Salzburg, Austria; Healthcare AG, Zurich, Switzerland; Department of Pharmaceutical Chemistry, College of Pharmacy, King Saud University, Riyadh 11451, Saudi Arabia

In the published article Calabrese V, Osakabe N, Khan F, Wenzel U, Modafferi S, Nicolosi L, Fritsch T, Jacob U.M., Abdelhameed A.S., Rashan L. Frankincense: A neuronutrient to approach Parkinson’s disease treatment. Open Med. (Wars) 2024; 19(1): 20240988, doi: 10.1515/med-2024-0988. PMID: 38911256 PMCID: PMC11193358, the affiliation of Ursula M Jacob is not correct.

The correct author affiliation is as follows:

Ursula M. Jacob: Healthcare AG, Zurich, Switzerland

